# In-Plane Rotation of Prolate Colloids Adhered to a Planar Substrate in the Presence of Flow

**DOI:** 10.1021/acs.langmuir.3c00433

**Published:** 2023-04-25

**Authors:** Ran Ran, Jianfeng Sun, Sinan Müftü, April Z. Gu, Kai-Tak Wan

**Affiliations:** † Department of Mechanical and Industrial Engineering, Northeastern University, Boston, Massachusetts 02115, United States; ‡ Department of Civil and Environmental Engineering, Cornell University, Ithaca, New York 14853, United States

## Abstract

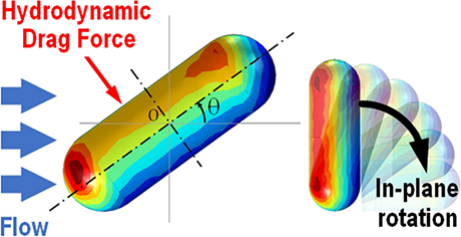

Micron-size spherical polystyrene colloidal particles are mechanically stretched to a prolate geometry with desirable aspect ratios. The particles in an aqueous medium with specific ionic concentration are then introduced into a microchannel and allowed to settle on a glass substrate. In the presence of unidirectional flow, the loosely adhered particles in the secondary minimum of surface interaction potential are easily washed off, but the remnant in the strong primary minimum preferentially aligns with the flow direction and exercises in-plane rotation. A rigorous theoretical model is constructed to account for filtration efficiency in terms of hydrodynamic drag, intersurface forces, reorientation of prolate particles, and their dependence on flowrate and ionic concentration.

## Introduction

Transport and adhesion of microbes through porous media (e.g., sand column) play crucial roles in water filtration and directly affect human health.^1^ The filtration process is a complex interplay of mechanisms such as particle coagulation, physical straining and clogging, and surface interactions.^2−4^ The underlying physics and mechanics of transport of colloidal particles through a porous medium have attracted extensive attention over the past decades. The classical colloidal filtration theory, addressing three dominant transport mechanisms, namely, interception, gravitational sedimentation, and diffusion, is essentially built upon the general convective-diffusion equation to predict filtration efficiency.^5,6^ Improved models consider the van der Waals (vdW) attractions and hydrodynamic drag on the deposited particle, as well as particle–particle and particle–collector interactions, to explain the influences of the environment, such as ionic concentration of the electrolytic medium and flow velocity.^7−10^

The Derjaguin–Landau–Verwey–Overbeek (DLVO) theory provides the basic theoretical tool to incorporate surface interactions into filtration.^11,12^ When a charged colloidal particle approaches a charged substrate, electrostatic double layers build up, leading to repulsion in addition to the intrinsic van der Waals attraction. The resulting intersurface potential is a short-range primary minimum (1-min) with strong attraction, an intermediate repulsive potential, and a long-range secondary minimum (2-min) with weak adhesion.^13^ An increase of ionic concentration in the electrolyte reduces the electrostatic repulsion and allows more particles to move from the weakly bounded 2-min to the strong 1-min. Other biochemical factors also influence the particle interaction with the substrate, e.g., bacterial motility, cell size, and cell wall stiffness.^14,15^ Our earlier model relates the inherent microscopic properties of a single bacterial cell with the macroscopic filtration process via a dimensionless Tabor’s parameter.^16,17^ A distinct shortcoming of the existing model is the assumption of bacterial cells only in a spherical shape or an average radius of non-spherical cells. The fact is that the geometrical skewness or eccentricity of ellipsoidal particles plays a crucial role in filtration.^18−20^ A slender cell makes a relatively larger intimate contact area with the substrate, but the streamlined body reduces the dynamic shear due to external flow and becomes less prone to detachment.

In this work, we investigate prolate polystyrene particles to simulate the common bacterial geometry. Spherical particles are mechanically stretched to a desirable aspect ratio,^21−23^ and a homemade microfluidic device is constructed to quantify filtration efficiency. To circumvent the complex clogging and straining and to emphasize particle–substrate interactions, a single, rather than multiple, layer of particles is allowed to settle on a glass substrate that simulates sand surfaces. The adhered particles are subject to a steady external flow of the aqueous medium. The particle–substrate intersurface interaction is accounted for by the DLVO theory, and hydrodynamic drag is derived from fluid mechanics using a multi-physics software COMSOL (v5.1, Stockholm, Sweden).^24,25^

## Materials and Methods

**Figure 1:**
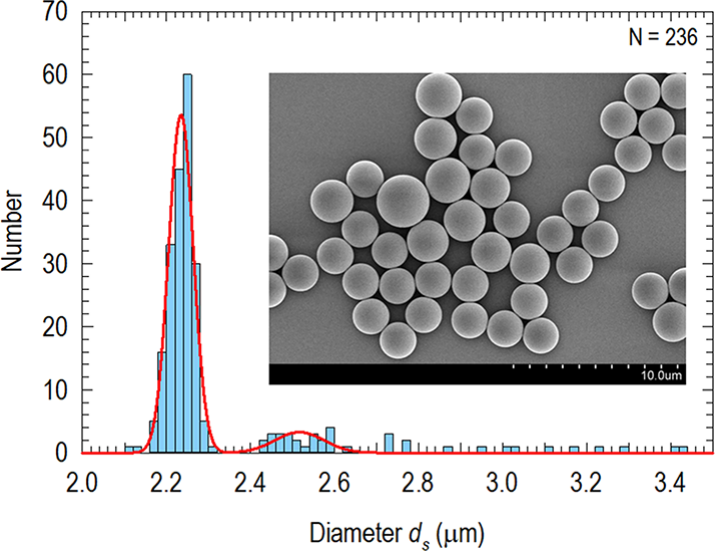
Intrinsic distribution of particle diameter with a main peak at 2.23 μm in a sample batch of 236 particles. The inset shows a scanning electron micrograph of typical polystyrene spheres.

Spherical polystyrene particles (Spherotech, Lake Forest, IL) with an average diameter of ∼2.29 μm, coated with palladium are imaged by a high-resolution field emission scanning electron microscope (Hitachi S-4800) at 3 kV. Figure 1 shows typical polystyrene spheres and the distribution of sphere diameter, *d*_s_. A major peak occurs at *d*_s_ = 2.23 ± 0.01 μm and a minor peak at *d*_s_ = 2.52 ± 0.01 μm.

**Figure 2:**
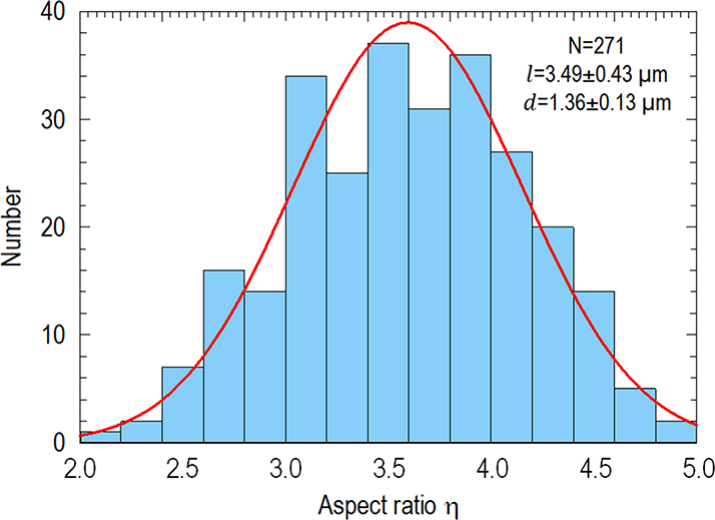
Statistical distribution of the aspect ratio η of mechanically stretched polystyrene spheres in a sample batch of 271 particles.

**Figure 3:**
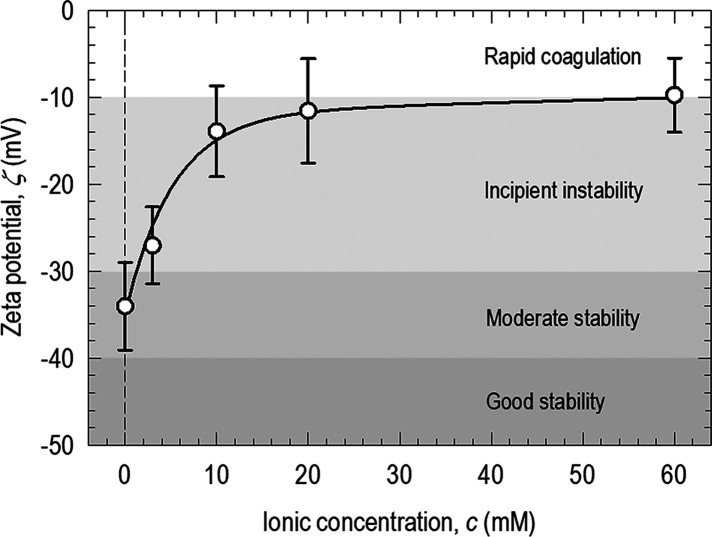
Zeta potential ζ (mV) of polystyrene particles as a function of the ionic concentration of the electrolyte. Beyond ∼20 mM, ζ reaches a plateau and becomes independent of *c*. A smooth curve is drawn to connect the data to show the trend.

To fabricate elongated particles, as received spherical particles are introduced into 5 wt % polyvinyl alcohol (MW 89,000–98,000, 99+% hydrolyzed, from Sigma-Aldrich) at a concentration of roughly 0.05% (wt/vol). Glycerol at ∼1% (wt/vol) is added as a plasticizer.^22^ The colloidal solution is air-dried in a petri dish to form a ∼70 μm thick film. A rectangular strip cut from the film is mounted onto a simple mechanical stretcher that can extend in the longitudinal direction.^21^ The strip is heated to ∼145 °C in a silicon oil bath and is stretched at a speed of ∼12 mm/min to the desired aspect ratio. The particles are retrieved by dissolving the film in a 30% isopropanol alcohol (IPA, I9030, Sigma-Aldrich), followed by centrifugation in a 30% IPA and deionized water. The elongated particles are characterized by optical microscopy. A standard MATLAB (version R2019a, MathWorks, MA) image processor is used to determine the aspect ratio of individual particles, η = (*l* + *d*)/*d*, with *l* the length of the cylinder and *d* the diameter of spherical caps. Figure 2 shows η = 3.60 ± 0.53 with *l* = 3.49 ± 0.43 μm and *d* = 1.36 ± 0.13 μm. Zeta-potential, ζ, of the stretched particles, is measured using a 90 Plus Particle Size Analyzer (Brookhaven Instruments Co., NY). Figure 3 shows the monotonically increasing negative zeta-potential, ζ(*c*), of the processed particle surface as a function of ionic concentration, *c*.

A microfluidic channel is fabricated as in our earlier work.^26^ The dimensions are representative of the volume between sand particles in a filtration column.^26^ In brief, a 1:10 precursor to polydimethylsiloxane (PDMS, SYLGARD 184 Silicone Elastomer, Dow Corning) is poured into a mold and cured at 69 °C for 2 h before being attached to a planar glass substrate to form a channel with height 70 μm, length 25.0 mm, and width 3.0 mm. Commercial glass slides are known to have surface roughness in the order of root mean square 0.2 μm, which is negligible compared to the particle diameter. The PDMS–glass interface is strengthened by heating at 85 °C for an hour. Two holes with size gauge 20 (diameter 0.908 mm) are made at opposite ends of the channel for liquid inlet and outlet, and an additional hole is made at the midspan to introduce particles. The assembly is placed on an inverted optical microscope for in situ observation and measurement. A syringe pump (model NE-300, New Era Pump System Inc., Farmingdale, NY) is used to drive potassium chloride, KCl (aq) into the channel from the inlet and drains at the outlet. The liquid in the channel becomes stagnant as the flow is halted. A small drop of KCl (aq) with a particle density of ∼5 × 10^5^ μL^–1^ is gradually injected via the midspan inlet to deposit a patch of particles on the glass substrate until the occupied area reaches a radius of ∼2 mm. The elongated particles then settle with the random orientation of their individual axes. In the presence of flow, particles will be washed down from the upstream. The edge of the circular patch close to the inlet, therefore, represents the area of interest, because there are no particles carried downstream blocking the view. An inverted microscope (GX 71, Olympus, Tokyo, Japan) focuses on this observation area and monitors the particles adhered to on the substrate. The spatial distribution of the particles is recorded before, during, and after the flow.

To investigate filtration behavior, the flowrate is increased in a stepwise manner from 0.037 to 37 mm/s, which is the typical velocity range of groundwater. An optical micrograph is taken initially at quiescence to record the total number, spatial distribution, and orientation of deposited particles. At *t* = 0, the flow is raised to and held at *V* = 0.037 mm/s until all loosely bounded particles are washed off and a steady state is reached. The flowrate is increased in a stepwise manner to *V* = 0.37, 3.7, and 37 mm/s, with a steady state reached at every interval. An alternative approach is to raise the flowrate directly from quiescence to a desirable value rather than the stepwise increase. In the latter approach, the sudden shock washes off more particles than the stepwise process but allows rapid measurements. To quantify filtration, an efficiency, 0 ≤ α ≤ 1, is conventionally defined to be the fraction of particles retained by the substrate or collector surface. The lower bound of α = 0 indicates all particles being washed off and the substrate failing to filter out any particles. The upper bound of α = 1 indicates retention of all particles and ideal filtration.

## Results

**Figure 4:**
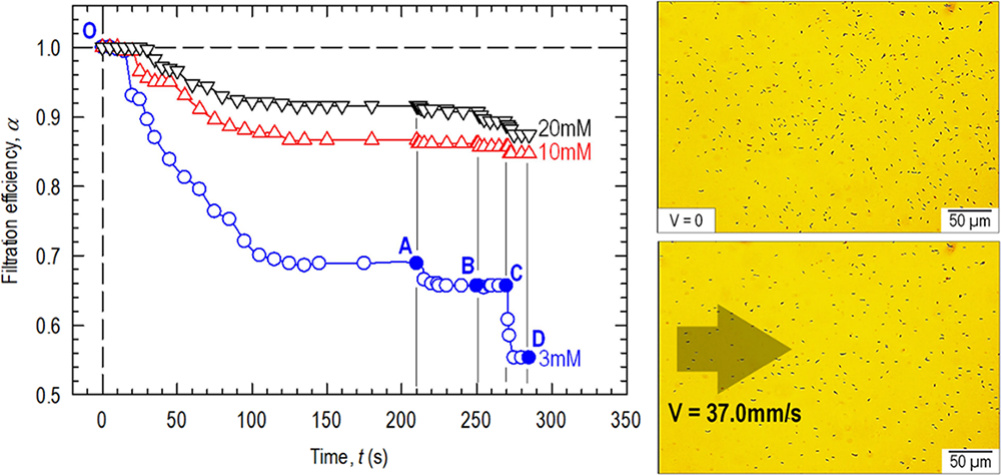
Temporal filtration behavior when particles are exposed to electrolytes with different ionic concentrations. In a 3 mM KCl(aq), the initial flow reaches a steady state at *t* ≈ 130 s and α reduces to a constant, prior to a stepwise increase in *V* at A, B, and C. Experiment is repeated at other concentrations. Optical micrographs show typical particle distribution at O (quiescent) and D (*V* = 37 mm/s, arrow shows flow direction).

At a specific ionic concentration *c* (KCl), a progressive stepwise increase in flowrate leads to characteristic filtration behavior. Figure 4 shows typical temporal behavior α(*t*). The baseline is set at the total number of particles settled *t* = 0, *V* = 0, and α = 1. At the onset of flow, the loosely adhered particles in 2-min are gradually washed off until a steady state is reached and α settles at a constant value. As *V* increases in a stepwise manner, more particles are removed. It is worthwhile to note several features of α(*t*): (i) the more concentrated the electrolyte, the higher the particle retention in the 1-min and thus higher α, (ii) at a higher flowrate, more particles are washed off and α diminishes. In the case of *c* = 3 mM and *V* = 0.037 mm/s, it takes roughly 130 s to remove the loosely bounded particles reaching a steady state with constant α. Increase in flowrate from A (*V* = 0.37 mm/s) to B (*V* = 3.70 mm/s) to C (*V* = 37 mm/s) left behind progressively less particles on the substrate. At higher *c*, changes in α(*t*) are less significant.

**Figure 5:**
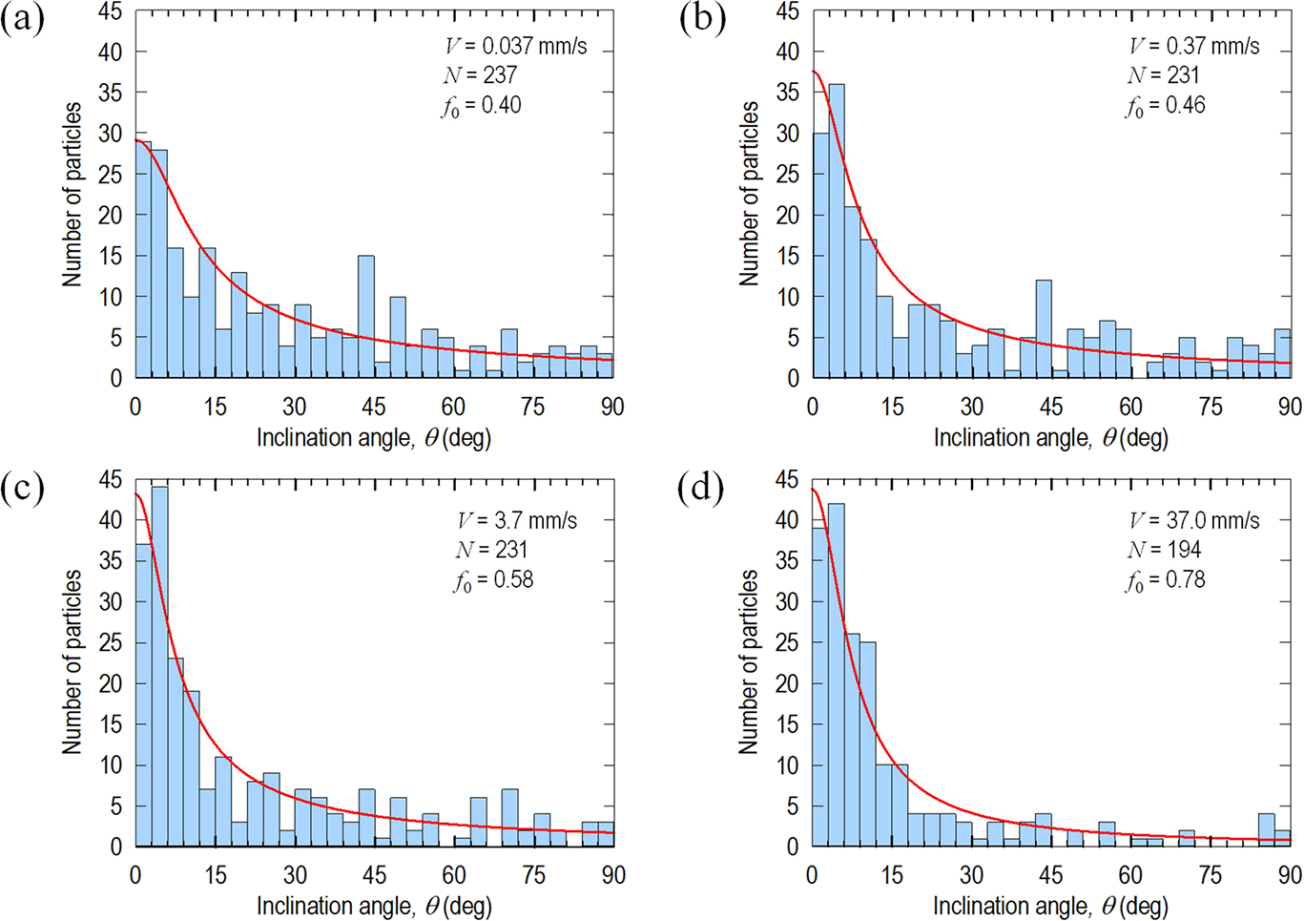
(a–d) Typical distribution of particle orientation at *c* = 3 mM. The higher the flowrate, the higher the tendency to align with flow. Flowrates are raised in a stepwise manner.

An interesting phenomenon is observed in addition to assessing the filtration behavior in terms of a number of particles. A significant proportion of the remnant particles is found to have one end stuck on the substrate while the other end is free to rotate to some extent. Depending on the flowrate, particles align partially with the flow direction subtending an angle θ to the flow direction, until they are washed off at a sufficiently high *V*. When θ = 0°, the particle is perfectly aligned with the flow direction. To quantify the degree of alignment in an ensemble,^27,28^ an orientation factor is defined as follows:
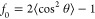
1with  averaged over the total number of particles, *N*. The lower bound of *f*_0_ = 0 corresponds to random orientation, and the upper bound of *f*_0_ = 1 indicates perfect alignment with the external flow. Figure 5a,d shows histograms of the number of particles inclined at an angle, θ, to the flow direction at A, B, C, and D (c.f., Figure 4). A higher *V* causes more particle alignment. Measurements are modeled by the *t* location-scale distribution with probability density function given by
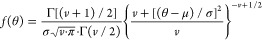
2with Γ the gamma function, μ the mean of distribution (μ = 0), σ the scale parameter, and ν the shape parameter.^29^ Curve-fitting yields (σ, ν) = (28, 0.1) for *V* = 0.037 mm/s, (18, 0.12) for *V* = 0.37 mm/s, (14, 0.15) for *V* = 3.7 mm/s, and (9, 0.5) for *V* = 37 mm/s.

**Figure 6:**
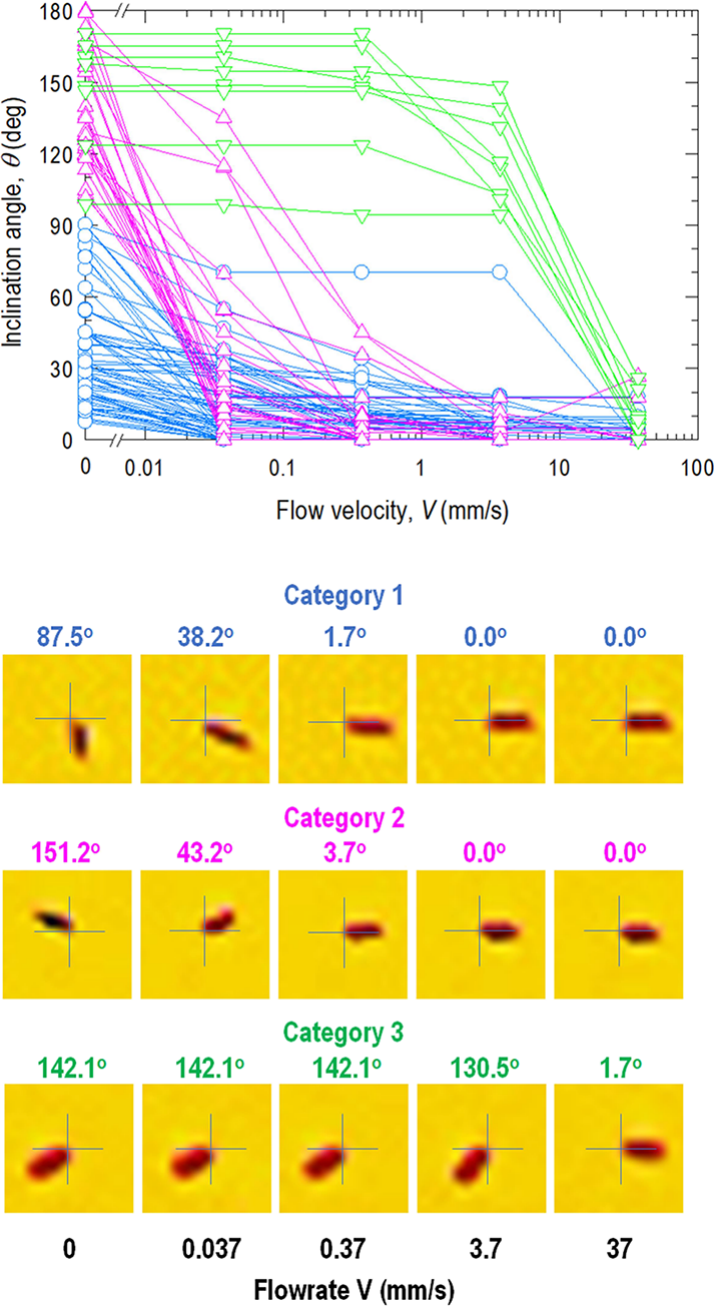
Tracking rotation as flow increases. In each category, images belong to the same particle at different flow velocities. Category 1 (blue) particles rotate by an acute angle as the frontal end is anchored. Category 2 (purple) particles rotate by an obtuse angle as the rear end is anchored. Category 3 (green) particles have the front end probabilistically detach from the substrate at critical *V*, and their axes thus move through an obtuse angle.

To better understand the stochastic behavior of reorientation, individual particles are monitored, resulting in a typical tracking map as shown in Figure 6. The seemingly confusing data can be classified into three types of rotation. In the first category, the prolate particle is initially inclined to the flow direction by 0° ≤ θ_0_ ≤ 90°, its front end facing the flow being strongly anchored to the substrate but its rear end relatively loosely bounded. Increasing *V* pushes the axis to align and to move through an acute angle 0° < Δθ < 90° such that the particle is perfectly aligned with θ = 0 at sufficiently large *V*. In the second category, the rear end is anchored, while the front end is relatively free to move. As *V* increases, the axis moves gradually through an obtuse angle of 90° < Δθ < 180°. A high flowrate leads to θ = 0°, which is indistinguishable from θ = 180° in an optical micrograph. The third category has a similar behavior as 90° < Δθ < 180°, but the particle holds on to its position until a critical *V* is reached triggering rotation. The particle is initially stuck on both ends on the substrate, then the front end gives way probabilistically, followed by a sudden rotation.

**Figure 7:**
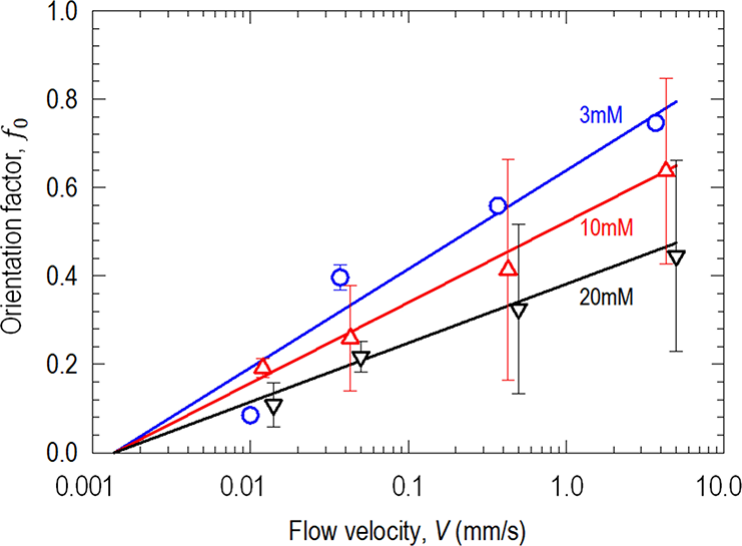
Degree of particle alignment as a function of flowrate in the presence of the electrolyte with a range of potassium chloride concentration. Every data point is obtained when the flowrate is increased to the desired value in one step. Data are slightly displaced laterally for clarity.

Figure 7 shows *f*_0_ as a function of *V* for three different *c* levels, for one step increase in *V* from quiescence to a range spanning *V =* 0.01 to 3.7 mm/s. At a steady state, a portion of particles is washed off and carried downstream while the rest remains adhered. For a specific *c*, *f*_0_ is proportional to log(*V*), consistent with the stepwise process. In qualitative terms, a higher *V* exerts a stronger rotational moment or torque on the particles and thus more alignment. A high ionic concentration strengthens the particle adhesion and thus a large inertia to rotate, resulting in larger *f*_0_. A phenomenological equation correlating *f*_0_, *c*, and *V* can be given as follows:
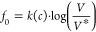
3where *k*(*c*) ≈ −0.00571*c* + 0.23714 and V* ≈ 1.37 × 10^–3^ mm/s by curve fitting. These constants have physical implications. For instance, V* marks the minimal flowrate below which the hydrodynamic shear is too small to cause any measurable alignment. The function *k*(*c*) implies a critical concentration of c* ≈ 40 mM corresponding to too strong an adhesion that the particles become immobile, which is consistent with zeta potential measurement (c.f., Figure 3).

Hydrodynamic forces acting on a prolate particle with length (*l* + *d*), oriented at an angle, θ, to the flow direction, and being adhered to a flat substrate are modeled numerically. The intrinsic intersurface interaction in an electrolyte is modeled by the DLVO theory.^24,30,31^ Electrostatic double layers on the surfaces create repelling forces, while van der Waals interaction causes attractive forces. The net attractive force, *F*_a_, depends on the zeta potential and ionic strength of the aqueous medium. Particles orthogonal to the flow with θ = 90° experience hydrodynamic drag that is proportional to the frontal area *A* = *l* × *d* + π*d*^2^/4, i.e., the rectangular central section and two polar caps, which can be measured using the optical micrographs. Our preliminary model of a cylinder adhering to a planar substrate with a line contact force^31^ is adopted in this work. Buoyancy and particle weight are neglected.

**Figure 8:**
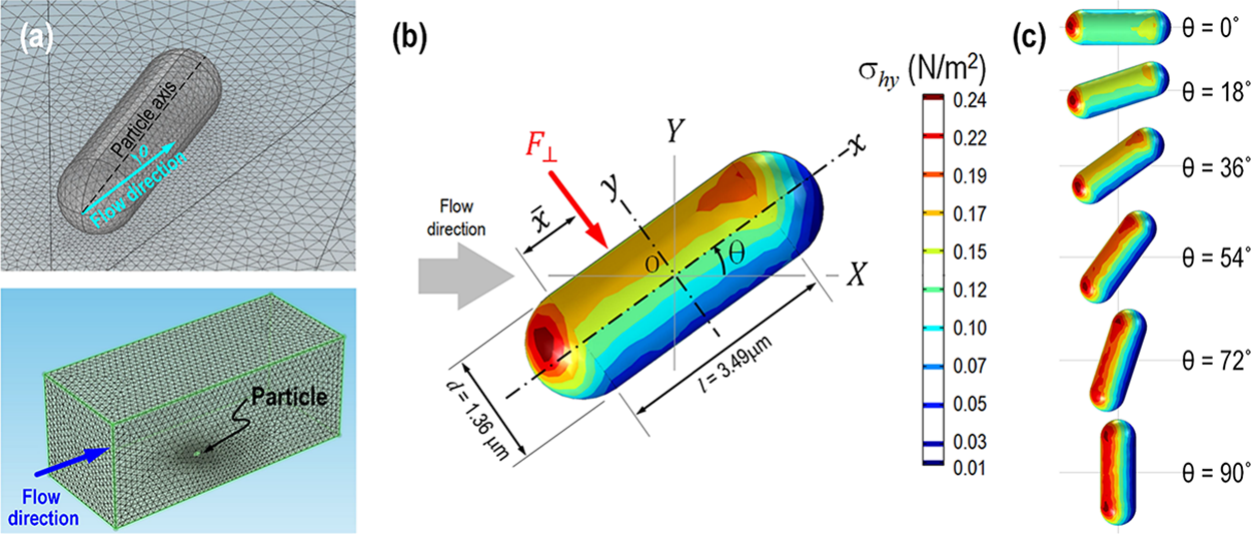
Prolate particle is subject to a flow *V* = 0.37 mm/s at an angle of θ to its axis. (a) Customized tetrahedral mesh around the prolate particle (top) and finite element mesh for the solution domain (bottom). (b) Hydrodynamics drag or von Mises stress on the particle surface, σ_*hy*_ with θ = 36°. (c) Hydrodynamic drag on a particle with a range of θ. It takes roughly 1.5–2 h to run a single test.

Flow around the prolate particle is modeled by using COMSOL (v.5.1, Stockholm, Sweden). Figure 8a shows the finite element meshes around the particle and the microchannel. The mesh size is raised gradually from 0.1 μm on the particle to 8 μm in a distance in order to resolve the rapid pressure and velocity gradients that occur near the particle. The element growth rate is set to 1.08, the curvature factor to 0.3, and the resolution of narrow regions to 0.95. The particle with dimensions of *l* = 3.49 μm, *d* = 1.36 μm, or an aspect ratio η = 3.60, inclines at an angle θ to the flow direction. The global reference frame is (*X*, *Y*, *Z*), while the local frame of the particle is (*x*, *y*, *z*). The particle rests on the *X–Y* plane of the substrate (*Z* = 0) with its axes along the *x* and *y* directions. The particle makes a line contact with the bottom surface of the channel. The net hydrodynamic force vector on the particle is given by
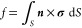
4with ***n*** the unit normal to the particle surface, *S* the total surface area of the particle, and **σ** the stress tensor. The velocity field at every point on the mesh is computed by the Navier–Stokes equations based on mass conservation for an incompressible fluid. The pressure and shear are computed at each point in the fluid, including the surface of the particle. Traction on the particle surface is given by
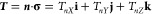
5where **i**, **j**, and **k** are the unit normal vectors in the *X*, *Y*, and *Z* directions, respectively.

Note that the traction components *T_nX_*, *T_nY_*, and *T_nZ_* are given in terms of the local stress components defined with respect to the Cartesian axes as follows:
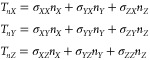
6

The net force on the particle found from eq 4 thus comprises the normal and shear stresses. Boundary conditions are given by setting: (i) the inlet flow rate to a desired value, e.g., *V* = 0.37 mm/s; (ii) the pressure at the microchannel outlet to ambient; and (iii) no-slip conditions on the surfaces of the particle and the channel. Lift force in the *Z*-direction is negligible compared to drag forces in the *X–Y* plane. The calculation is repeated for θ = 0° to 90° with an increment of 3°. Flow exerts a 2D hydrodynamic drag on the particle. The resultant force has components *F*_∥_ and *F*_⊥_ parallel and perpendicular to the particle axis, respectively, in that 
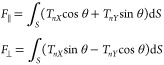
7

Hydrodynamic drag over the particle surface is given by 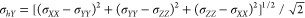


Drag is maximum at the frontal area and minimum at the leeside. Figure 8b shows a particle with θ = 36°, and Figure 8c shows a particle for a range of θ. Net pressure integrated over the entire particle surface yields the net hydrodynamic drag. The resultant force *F*_⊥_ acts at a location (*x̅*, *z̅*) along the particle axis, where
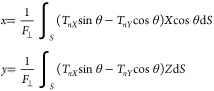
8which does not coincide with the centroid because of the asymmetric stress.

In Figure 8b with θ = 36°, the resultant force acts at a distance *Z̅* = 0.7*d* above the *X–Y* plane, leading to net moments of *M*_||_ = *F*_||_ × *Z̅* and *M*_⊥_ = *F*_⊥_ × *Z̅*. When *M*_||_ ≥ (*M*_||_)* = *F*_a_ × *l*/2, the particle tumbles about the *y* axis such that rotation is in the *x–z* plane. When *M*_⊥_ ≥ (*M*_⊥_)* = *F*_a_ × *a*, where *a* is half of the rectangular contact area width, the particle rotates about the *x* axis and rolls on the *x–y* plane. Particles perfectly aligned with the flow (θ = 0) experience negligible drag because of the streamlined geometry. Since *l* ≫ *a*, (*M*_||_)* ≫ (*M*_⊥_)* rolling will occur prior to tumbling. It is, however, difficult to observe rolling and tumbling with the optical microscope, since that marks the onset of detachment from the substrate.

**Figure 9:**
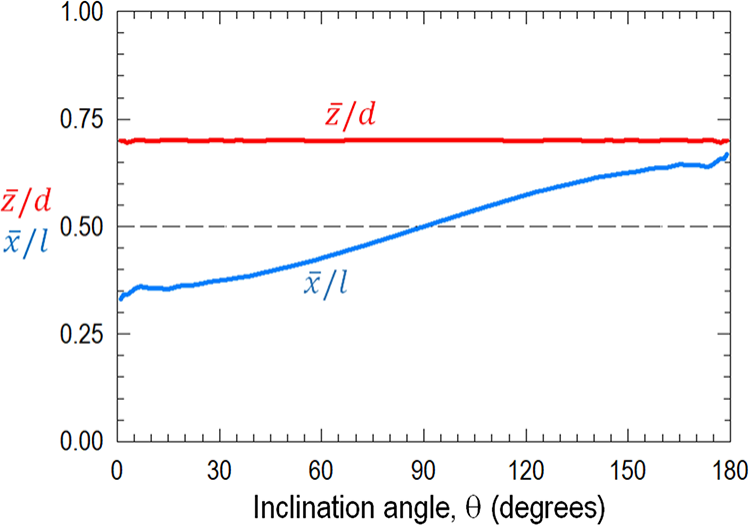
Computed rotation moment arm *x̅*/*l* of net torque on a particle anchored at one end monotonically increases with the angle θ inclining to the flow direction. The computed rolling moment arm is found to be *z̅* ≈ 0.7*d* almost independent of θ. The dashed line at *x̅*/*l*=0.5 and *z̅*/*d*=0.5 denotes the axes of symmetry.

One end of the particle can be the anchor point due to surface charge heterogeneity and non-uniform roughness of the particle or substrate. The hydrodynamic torque to drive an in-plane rotation from θ_0_ to θ is given by *M*_R_ = *F*_⊥_ × *x̅*, with *x̅*(θ) the moment arm from the anchor to the net force *F*_⊥_(θ), which is the pressure integrated over all particle surface. Since the particle is anchored at one end, *F*_⊥_ does not act at the midspan of the particle but skews toward the anchor point. For θ = 0°, the particle is aligned with the front end anchored. The resultant force thus acts at the centroid of the streamlined body with *x̅* = 0 and no rotation occurs as long as the anchor is sufficiently strong. As θ rises from 0° to 90°, *x̅* increases and so do *F*_⊥_ and *M*_R_. Figure 9 shows the increasing moment arm *x̅* from θ = 0° to 180°, which depends only on particle geometry.

**Figure 10:**
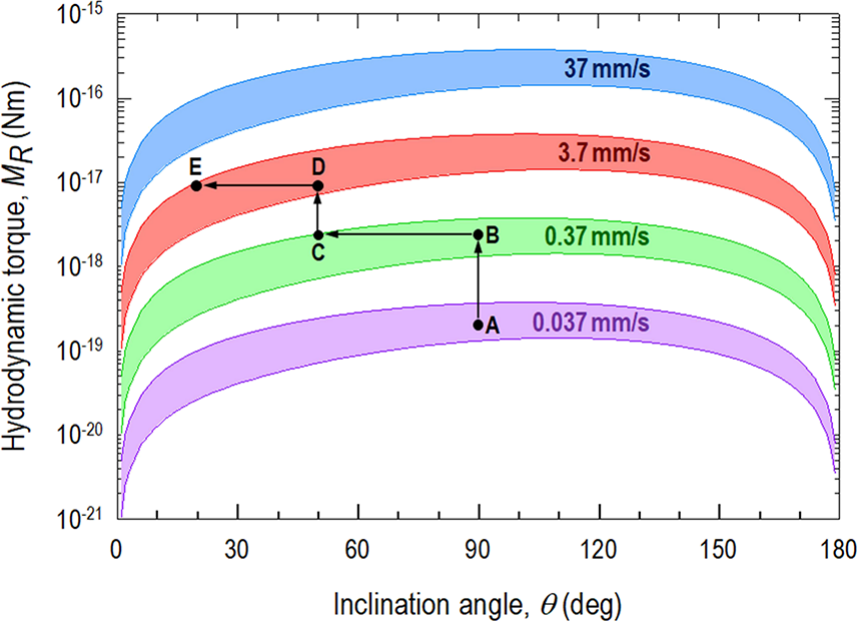
Hydrodynamic torque on a particle with *l* = 3.49 ± 0.43 μm and *d* = 1.36 ± 0.13 μm in *c* = 3 mM KCl (aq) as a function of inclination angle to the flow direction. Flowrate follows a stepwise increase to a final *V* = 37 mm/s. Rotation follows ABCDE (see text).

Figure 10 shows the resultant torque *M*_R_(θ) on the particle for a range of flow velocities. It is noted that *M*_R_ (θ = 0°) = *M*_R_ (θ = 180°) = 0 because of symmetry. *M*_R_(θ) appears as bands due to the inevitable stochastic variances of particle size and aspect ratio. The larger and the longer the particles, the higher the hydrodynamic drag. At a specific *V*, *M*_R_ reaches its maximum at θ = 105°, before monotonically diminishing and finally vanishing at 180°. Increase in *V* raises *M*_R_ significantly. Rotation ceases when the external torque is supported by the moment due to intersurface attraction. As a hypothetical example, the rotation of a specific particle originally inclining at θ = 90° follows path ABCDE. At A, *V* is slow and the torque is low, and the particle remains at a steady state. When flow increases to 0.37 mm/s at B, the drag overcomes the intersurface attraction *M*_R_ > *M*_R_* and is sufficient to rotate the particle to C where θ decreases to 50°. However, the moment arm and thus *M*_R_ diminish along BC (c.f., Figure 9), and the particle is once again stuck until *V* reaches 3.7 mm/s at D and the particle rotates to E with θ ≈ 20°. The particle grows progressively more aligned with the flow along ABCDE. In reality, particle rotation likely follows a smooth trajectory rather than a stepwise increase.

## Discussion

It is interesting to investigate how morphology influences the fate of adhered particles in the presence of flow. When particles come to close proximity to a glass substrate, the intersurface forces depend on the combined effects of the van der Waals attraction and electrostatic double-layer repulsion.^26,31^ According to the classical DLVO model, the potential energy between a spherical particle and a planar substrate with a separation, *z*, can be written in the simplest form
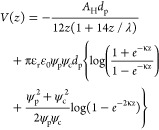
9with *d*_p_ being the average particle diameter, *A*_H_ being the Hamaker constant, λ being the characteristic length of the dielectric, ε_r_ being the dielectric constant of water, κ being the reciprocal of Debye length, and ψ_p_ and ψ_c_ being the surface potentials of the particle and substrate, respectively. The first negative term in eq 9 denotes the van der Waals attraction, and the second positive term denotes the electrostatic repulsion. In essence, *V*(*z*) comprises a short-range strong primary potential minimum (1-min) and a long-range weak secondary potential minimum (2-min) separated by a repulsive energy barrier. Eq 9 applies to the adhesion of a soft cylindrical shell on a planar substrate with elaborate modifications.^32^ In the present context, the prolate polystyrene particles are stiff and virtually rigid with respect to the weak hydrodynamic drag that the mechanical deformation is minimal and eq 9 is adequate to describe the adhesion behavior. This is consistent with the classic work in comparing spherical and cylindrical particles.^33^

Particles on the substrate surface thus stochastically fall into 1- or 2-min. In the presence of flow, a hydrodynamic drag develops to detach the particles. For a typical ensemble of particles, the frontal area and aspect ratio of individual particles are measured from the optical micrographs using the aforementioned MATLAB image processor. The occurrence of the three different fates of particles, namely, remaining stationary or stuck on the substrate surface, detached and removed by the channel flow, and reorientated. Table 1 shows typical data collected in a stepwise flow test at *c* = 3 mM.

**Table 1:** Fate of Particles Initially Deposited on the Glass Substrate at *c* = 3 mM^a^

category		frontal area A (×10^–12^ m^2^)	aspect ratio η	occurrence
stationary		6.73 ± 2.38	3.54 ± 0.91	8.6%
detachment and removal		4.97 ± 1.61	3.01 ± 0.78	43.5%
reorientation	rotation about rear anchor	5.73 ± 3.03	3.07 ± 1.07	17.2%
	rotation about front anchor	6.06 ± 3.17	3.20 ± 0.89	3.7%

a Flowrate is similar to that shown in Figure 4.

In general, it is reasonable to assume that the particles that remain stationary are those that are trapped in the 1-min of the surface potential and thus develop a strong adhesion and firmly adhere to the substrate. These particles are comparatively large in dimension and have therefore large contact area with the substrate. Their high η leads to a streamlined body experiencing smaller hydrodynamic drag. Particles being detached are those that are in the 2-min and are bonded relatively loosely to the substrate. They are small and rounded with small η. At a sufficiently high flowrate, they are removed from the substrate. The rest of the particles reorient to align with the flow direction.

**Figure 11:**
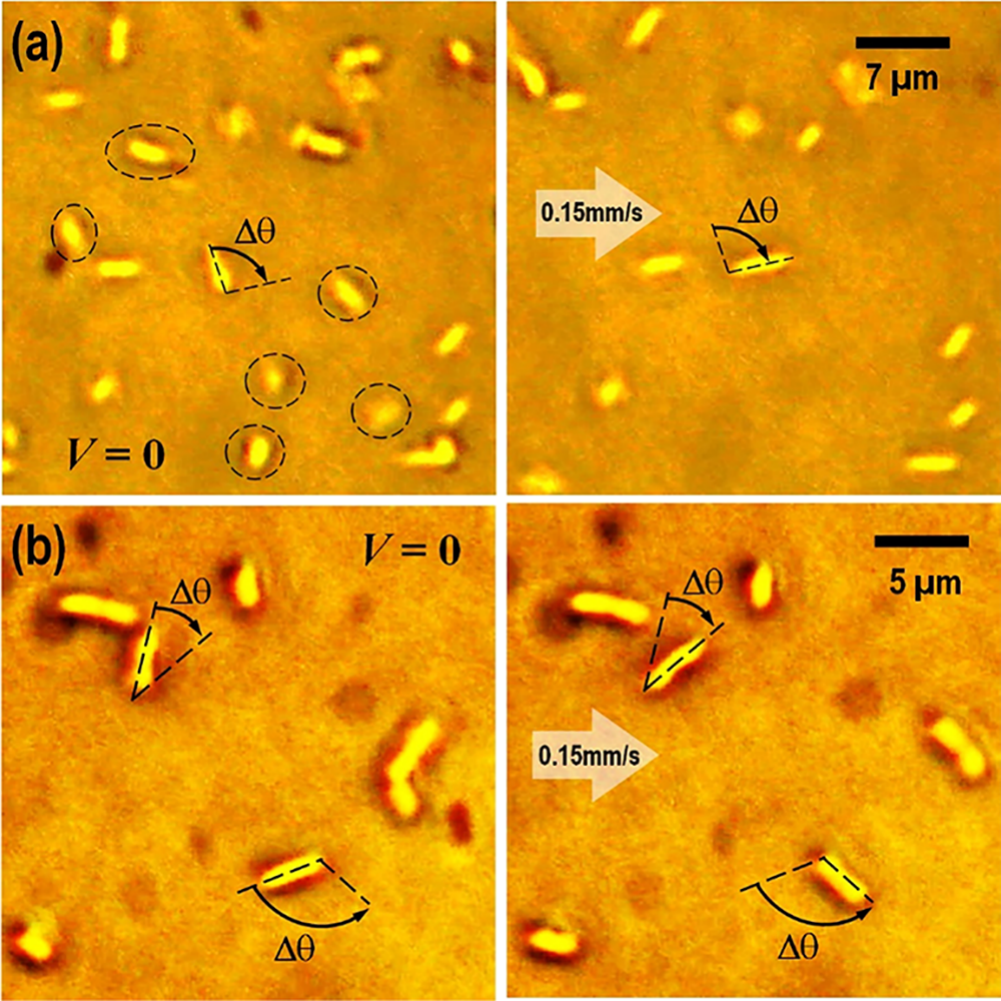
(a) Strain A and (b) strain H deposited on the glass substrate are exposed to 3 mM KCl (aq) and subject to *V* = 0.15 mm/s. Particles are observed to (i) remain stationary, (ii) detach and be removed (circled by dashed lines), or (iii) be reorientated with Δθ. Particles with frontal anchors rotate through an acute angle and those with rear anchors through an obtuse angle. Arrows show flow direction.

Considering flow velocity and surface forces being likely to play major roles in the interaction of bacteria attached to a surface and subjected to external flow, it is reasonable to expect bacteria in general to interact with the flow in ways similar to the elongated polystyrene particles. As a demonstration, two common rod-shaped bacterial strains, namely, *Bacillus cereus* (strain H) with *d*_H_ = 0.73 ± 0.06 μm, *l*_H_ = 2.16 ± 0.31 μm, and η_H_ = 2.95 ± 0.18, and *Raoultella ornithinolytica* (strain A) with *d*_A_ = 0.8 ± 0.08 μm, *l*_A_ = 1.58 ± 0.40 μm, and η_A_ = 1.98 ± 0.25, are tested in the microfluidic channel using the aforementioned procedure.^16,17^ These strains are also characterized by an atomic force microscope (Agilent 5500, Keysight Technologies, Santa Rosa, California, US), giving modified Tabor’s parameter μ_H_ = 39.3 ± 3.3 and μ_A_ = 11.4 ± 0.41. This indicates strain H to be more adhesive than strain A. Figure 11 shows measurements showing stationary, detachment, and reorientation of the cylindrical strains as a result of flow and the associated hydrodynamic torque.

As a last remark, clogging of particles in micropores recently generates much interest in the colloidal community.^34−36^ In the present work, we focus on the in-plane rotation of adhered prolate particles subject to flow rather than coalescence. In fact, we ensure that multi-particle aggregates do not occur in the dilute solution so that particle reorientation can be observed and counted with minimal uncertainty.

## Conclusions

When colloidal particles in an electrolyte pass through a porous medium, the collector surfaces act as a trap via interfacial adhesion that depends on the ionic concentration. Liquid flow causes the particles to fall into different fates. The weakly interacting particles are detached and removed, while those firmly stuck determine the filtration efficiency. Depending on the flowrate and on how these particles are stuck, rotation on the collector surface is observed and is demonstrated by polystyrene particles and common bacterial strains. Bacterial alignment to the flow direction has significant consequences on the migration of deposited bacterial strains and the subsequent growth pattern when they multiply over time.

## Supporting Information Available

Mesh parameter; hydrodynamic forces; hydrodynamic stress; and normalized location (PDF)
