# Supplementary material for: In-Plane Rotation of Prolate Colloids Adhered to a Planar Substrate in the Presence of Flow
Source: Langmuir. 2023 Apr 25;39(18):6487–94. doi: 10.1021/acs.langmuir.3c00433 (PMC10620997; doi:10.1021/acs.langmuir.3c00433)
Supplement: Supplementary file 1 — la3c00433_si_001.pdf [file la3c00433_si_001.pdf]

Supporting Information

Figure 8a shows the customized tetrahedral mesh with the related parameters listed in **Error! Reference source not found.**S1. Velocity field is computed by using the Navier-Stokes equation and mass conservation for an incompressible fluid.

Predefined mesh size	Fluid dynamics – extra fine
Maximum element size	8.0 μm
Minimum element size	0.1 μm
Maximum element growth rate	1.08
Curvature factor	0.3
Resolution of narrow regions	0.95

Table S1. Mesh parameter used in simulation.

Table S2 lists the hydrodynamic force on a particle with l = 3.49 μm, d = 1.36 μm and η = 3.6 in the X, Y, Z directions using Equation S3 as a function of the inclination angle θ. Lift force in the Z-direction is negligible compared to drag forces in the X-Y plane. Figure S2 shows the net hydrodynamic stress over a particle surface with the stress levels for a range of misorientation angle θ between the cylinder and flow direction. Increasing θ from 0° to 90° raises hydrodynamic drag on the cylindrical surface.

Table S3 shows location where the resultant force $F_{\perp}$ acts as if it is a concentrated load as a function of θ. The distance $\bar{x}$ is measured from the anchored front end and is normalized to $\bar{x}/l$ by the major axis of the cylinder, while the distance $\bar{z}$ from the contact interface with substrate and is normalized to $\bar{z}/d$ by the diameter of the spherical cap. It is not possible to have θ = 0°, because of the geometrical obstruction due to the spherical caps. It is obvious that the frontal cylindrical pole is the stagnant point where the pressure is maximum and the streamlined body experiences the least drag. For θ ~ 3°, $\bar{x}/l$ approaches the limit of ~0.342 or $\bar{x} \approx 0.158\ l$ from the cylinder center corresponding to the longest moment arm. As θ increases to 90°, $\bar{x}/l$ is monotonic increasing and reaches 0.50 at θ = 90° where the hydrodynamic drag is maximum. If it is the rear end being anchored, $\bar{x}/l$ increases further to ~0.7 at 180°. Note that $\bar{z}/d \approx 0.7$ is virtually a constant at any θ.

θ (deg)	F_X (×10^{-13} N)	F_Y (× 10^{-13} N)
0	-7.49	-0.00976
3	-7.48	0.225
6	-7.54	0.430
9	-7.58	0.629
12	-7.66	0.834
15	-7.76	1.01
18	-7.91	1.18
21	-8.01	1.36
24	-8.17	1.51
27	-8.33	1.65
30	-8.52	1.78
33	-8.69	1.85
36	-8.91	1.95
39	-9.05	1.99
42	-9.27	2.01
45	-9.51	2.04
48	-9.71	2.02
51	-9.93	1.99
54	-10.1	1.94
57	-10.3	1.86
60	-10.5	1.75
63	-10.7	1.64
66	-10.9	1.50
69	-11.1	1.38
72	-11.1	1.21
75	-11.3	1.03
78	-11.4	0.819
81	-11.4	0.641
84	-11.5	0.425
87	-11.6	0.205
90	-11.6	-0.00324

Table S2. Hydrodynamic forces in X and Y directions acting on a cylindrical particle (c.f. Figure S1) as θ changes from 0˚ to 90˚ at $V = 0.37$ mm/s. Note that all computed $|F_Z| < 10^{-15}$ N appear as noise and show no consistent trend.

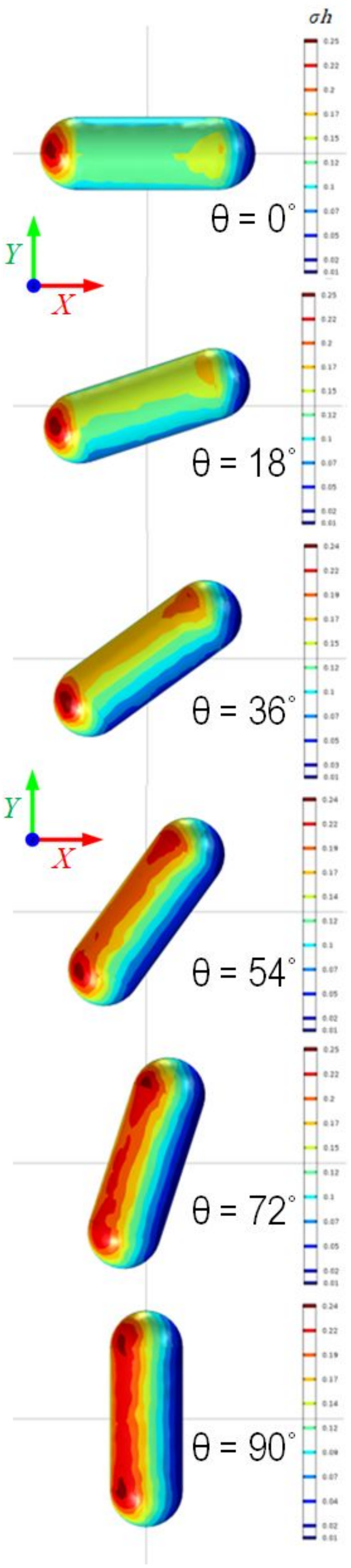

Figure S2. Hydrodynamic stress σ_{hy} acting on the cylindrical particle at a range of angle θ from the flow direction. Flow velocity is kept at V = 0.37 mm/s. Color code shows the stress level. Traction is minimum at θ = 0° because of the streamlined geometry, and maximum at θ = 90° as the cylinder appears as blunt object against the flow.

θ (degree)	$\bar{x}/l$	$\bar{z}/d$
0	-	0.827
3	0.342	0.696
6	0.359	0.702
9	0.358	0.700
12	0.357	0.700
15	0.355	0.701
18	0.361	0.701
21	0.363	0.700
24	0.366	0.701
27	0.371	0.701
30	0.374	0.701
33	0.377	0.700
36	0.382	0.701
39	0.385	0.700
42	0.391	0.700
45	0.396	0.701
48	0.402	0.701
51	0.408	0.701
54	0.414	0.701
57	0.419	0.700
60	0.427	0.700
63	0.434	0.700
66	0.440	0.700
69	0.448	0.701
72	0.455	0.701
75	0.462	0.701
78	0.470	0.701
81	0.477	0.701
84	0.485	0.701
87	0.492	0.701
90	0.500	0.701

Table S3. Normalized location of $F_{\perp}$ in x and z directions on a cylindrical particle as θ changes from 0˚ to 90˚.
